# Epidemic Risk from Cholera Introductions into Mexico

**DOI:** 10.1371/currents.outbreaks.c04478c7fbd9854ef6ba923cc81eb799

**Published:** 2014-02-21

**Authors:** Sean M. Moore, Kerry L. Shannon, Carla E. Zelaya, Andrew S. Azman, Justin Lessler

**Affiliations:** Department of Epidemiology, Johns Hopkins Bloomberg School of Public Health Baltimore, Maryland, USA; Department of International Health, Johns Hopkins Bloomberg School of Public Health, Baltimore, Maryland, USA; Johns Hopkins University, School of Medicine, Baltimore, Maryland, USA; Department of Epidemiology, Johns Hopkins Bloomberg School of Public Health Baltimore, Maryland, USA; Department of Epidemiology, Johns Hopkins Bloomberg School of Public Health Baltimore, Maryland, USA; Department of Epidemiology, Johns Hopkins Bloomberg School of Public Health Baltimore, Maryland, USA

**Keywords:** cholera

## Abstract

Stemming from the 2010 cholera outbreak in Haiti, cholera transmission in Hispaniola continues with over 40,000 cases in 2013. The presence of an ongoing cholera outbreak in the region poses substantial risks to countries throughout the Americas, particularly in areas with poor infrastructure. Since September 9, 2013 nearly 200 cholera cases have been reported in Mexico, as a result of introductions from Hispaniola or Cuba. There appear to have been multiple introductions into Mexico resulting in outbreaks of 2 to over 150 people. Using publicly available data, we attempt to estimate the reproductive number (R) of cholera in Mexico, and thereby assess the potential of continued introductions to establish a sustained epidemic. We estimate R for cholera in Mexico to be between 0.8 to 1.1, depending on the number of introductions, with the confidence intervals for the most plausible estimates crossing 1. These results suggest that the efficiency of cholera transmission in some regions of Mexico is near that necessary for a large epidemic. Intensive surveillance, evaluation of water and sanitation infrastructure, and planning for rapid response are warranted steps to avoid potential large epidemics in the region.

## Introduction

From 2001 to 2010, Mexico reported two or fewer cholera cases a year to the World Health Organization (WHO), and the region of the Americas as a whole reported no more than 36 cases a year between 2002 and 2009.^1^ Beginning in 2010 a large cholera outbreak began in Haiti, which subsequently spread to the Dominican Republic and Cuba. The Americas reported 361,266 cases in 2011, and 120,433 in 2012; and until recently cases had been largely confined to these three island nations.^1^ However, given frequent travel, ongoing introductions of cholera into other countries in the region are likely, and in September 2013 cases began to appear in Mexico harboring bacteria with a genetic profile 95% similar to* Vibrio cholerae* currently circulating of Haiti, Dominican Republic and Cuba.^2^

From September 9, 2013 to January 25, 2014 Mexico reported 185 confirmed cases of cholera caused by the toxigenic O1 El Tor strain in patients ranging from 3 months to 88 years old with one death (Figure 1).^2^^,^ 3 ^,^ 4 Additionally, two cases appeared in the Federal District in August, although reports indicate these were of a different, non-toxigenic strain.^5^ The first week of the current outbreak began with eight confirmed cases in Hidalgo State. The epidemic then rapidly exploded in Hidalgo, which had reported 160 confirmed cases by November 23. The majority of cases have occurred in the city of Huejutla de Reyes (population 115,000) in the Huasteca region of the state. Within Huejutla, the neighborhoods of Oxmal I, II, and III near the Tecoluco River have had the greatest number of cases, and the Mexican government has confirmed that the Tecoluco River was contaminated.^6^^,^ 7 Many residents in neighborhoods near the Tecoluco use the river to wash food and clothes, as well as for personal hygiene.^8^ Nearby states of San Luis Potosi, Veracruz and Mexico have had a smattering of cases, but to date these have not led to any other large outbreaks.^3^

**Map of confirmed cholera cases.**
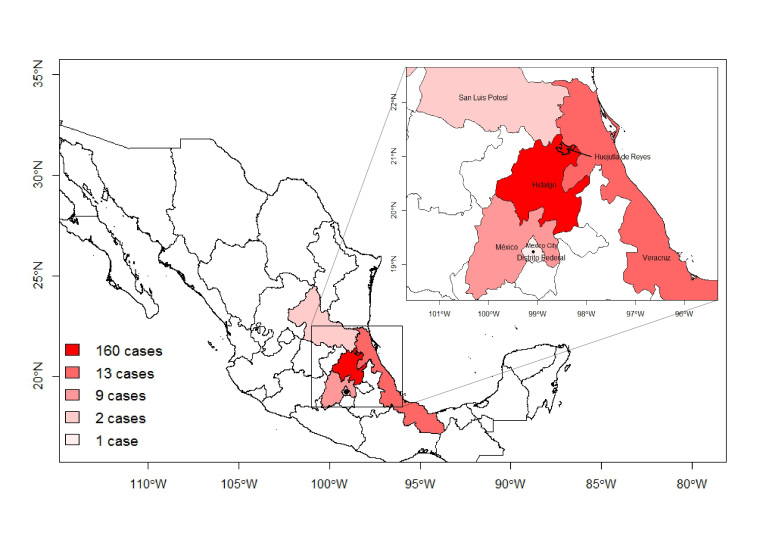
Map of confirmed cholera cases in Mexico by state since 9/1/2013. Does not include the two cases in Federal District from August, 2013.

Shortly after the start of the outbreak, the Mexican government began to implement a national prevention campaign including information dissemination strategies, follow-up investigations to identify potential secondary cases, the distribution of oral rehydration salt packages and chlorine tablets, and the liming of latrines, septic tanks, and the banks of the Tecoluco River in Huejutla, Hidalgo.^9^

Here we use published data to assess the efficiency of cholera transmission in Mexico as characterized by the reproductive number, *R* (i.e., the number of additional cases expected to be caused by each infected individual). If *R* is greater than one, then sustained cholera transmission is possible in Mexico, while if *R* is less than one, clusters of cases may still occur but a major epidemic or the endemic establishment of *V. cholerae* without an environmental reservoir could not happen. An estimate of *R* from this small outbreak could help public health authorities assess the likelihood of a larger epidemic spreading within Mexico or to other countries in the region.

## Methods

Identifying Likely Introductions

We estimated* R* from the distribution of the final sizes of suspected transmission chains stemming from likely independent introductions of cholera into Mexico during the outbreak.^10^^,^ 11 ^,^ 12 Through January 25, 2014 there have been 185 confirmed cholera cases (excluding 2 cases from Federal District in week 36 of 2013 that were of a different strain). Each cholera case was laboratory-confirmed as the O1 El Tor strain. Using publicly available data to evaluate how many independent introductions could have resulted in the observed clusters of cases, we considered four different scenarios: (1) a conservative estimate for R that assumes as many separate introductions and transmission chains as is reasonable (maximum introductions), (2-3) two likely scenarios based on the observed data and assumptions about the length of the generation interval for cholera (likely introductions), and (4) a scenario that assumes the fewest introductions and transmission chains possible (minimum introductions). Scenarios 1 and 4 are intended to put lower and upper bounds on plausible estimates of R.

**Table 1:** Summary of weekly confirmed cases in Mexico by state for 2013-2014. The first two cases in Federal District were a different strain than the rest of the cases.

*Week*	*Federal District*	*Hidalgo*	*Mexico State*	*San Luis Potosi*	*Veracruz*	*Total*
8/25/2013 – 8/31/2013	2	0	0	0	0	2
9/1/2013 – 9/7/2013	0	0	0	0	0	0
9/8/2013 – 9/14/2013	0	0	0	0	0	0
9/15/2013 – 9/21/2013	0	8	0	0	0	8
9/22/2013 – 9/28/2013	0	36	0	0	0	36
9/29/2013 – 10/5/2013	0	101	9	1	2	113
10/6/2013 – 10/12/2013	0	12	0	0	0	12
10/13/2013 – 10/19/2013	0	0	0	1	4	5
10/20/2013 – 10/26/2013	0	0	0	0	0	0
10/27/2013 – 11/2/2013	0	2	0	0	2	4
11/3/2013 – 11/9/2013	0	0	0	0	0	0
11/10/2013 – 11/16/2013	0	1	0	0	3	4
11/17/2013 – 11/23/2013	0	0	0	0	0	0
11/24/2013 - 11/30/2013	0	0	0	0	0	0
12/1/2013 - 12/7/2013	0	0	0	0	0	0
12/8/2013 - 12/14/2013	1	0	0	0	2	3
12/15/2013 - 12/21/2013	0	0	0	0	0	0
12/22/2013 - 12/28/2013	0	0	0	0	0	0
12/29/2013 - 1/4/2014	0	0	0	0	0	0
1/5/2014 - 1/11/2014	0	0	0	0	0	0
1/12/2014 - 1/18/2014	0	0	0	0	0	0
1/19/2014 - 1/25/2014	0	0	0	0	0	0
**Cumulative**	**2**	**160**	**9**	**2**	**11**	**187**

During the week of September 15, the first 8 cases were confirmed in 4 different municipalities within the state of Hidalgo (Table 1). Three of these municipalities (Tula, Ajacuba, and San Agustín Tlaxiac) are located near the capital city of Pacucha, while the fourth municipality, Huejulta, is several hundred kilometers away. It is not known whether all (or any) of these cases arose from a single primary infection or whether multiple introductions occurred. In the “maximum introductions” scenario we assume that each of the eight cases was a primary infection that started a new transmission chain. Under each of the “likely introductions” scenarios we assume that there were two introductions, one in Huejutla and another in the municipalities near Pachucha. For the fourth scenario (“minimum introductions”) we assume that there was a single introduction at the start of the outbreak. In the following week there was one case in Pachucha, Hidalgo and the other 35 cases were all in Huejutla. Under the “maximum introductions” scenario the case in Pachucha was a new primary case, but for the other scenarios we assume it was a secondary case. Starting the following week almost all of the cases in Hidalgo State were in Huejutla, therefore we assume a single transmission chain in Huejutla from week 38 until no cases were reported in week 42 for all scenarios.

In the “likely introductions” scenarios and the “minimum introductions” scenario we consider each introduction into a new state as the start of a new transmission chain. For our “maximum introductions” scenario we consider all cases during the first week in a new municipality to be co-primary and the start of new transmission chains. Confirmed cases following a week without any cases are considered new primary cases in the maximum introduction scenarios (although the generation interval for cholera can be greater than one week). For example, the cases in week 44 in Hidalgo and Veracruz that followed a week with no cases were considered to be the start of new transmission chains for this scenario. For the two "likely introductions" scenarios we assume a maximum generation interval of either one or two weeks. Confirmed cases occurring in a municipality after a gap in cases of either one or two weeks are assumed to be the start of a new transmission chain. All cases in the same and subsequent weeks (without interruption) in the same municipality are assumed to be from the same transmission chain. For the “minimum introductions” scenario we expanded the maximum generation interval to five weeks so that cases occurring in a state between one and four weeks after the last confirmed case were assumed to be secondary cases rather than new introductions. Using these assumptions we estimate that there have been a minimum of 5 (chains of 1, 2, 9, 13, and 160 cases) and a maximum of 37 (36 chains of one case, 1 chain with 149 cases) distinct transmission chains ranging in size from 1 to 160 cases. Under the most likely scenarios there have been either 8 transmission chains (1, 2, 2, 3, 6, 9, 11, and 151 cases) with a mean chain size of 23.1 cases and a mean for chains of size >1 of 26.3 cases or 13 transmission chains (1, 1, 1, 1, 2, 2, 2, 2, 3, 4, 6, 9, and 151 cases) with a mean chain size of 14.2 cases and a mean for chains of size >1 of 20.1 cases (Figure 2)

**Observed frequency distribution of outbreak sizes.**
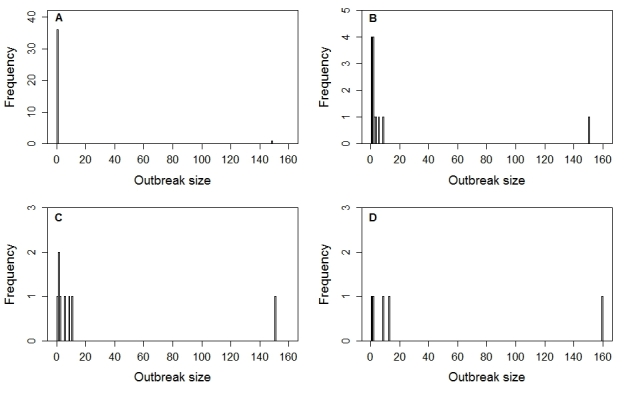
The observed frequency distribution of final outbreak sizes for cholera in Mexico in 2013-2014 for (a) a maximum of 37 introductions (outbreaks), (b) a likely scenario of 13 introductions with a maximum generation interval of one week, (c) a likely scenario of 8 introductions with a maximum generation interval of two weeks, or (d) a minimum of 5 introductions.

Estimating R from final size distribution

The transmission dynamics of individual cholera transmission chains were modeled using branching process theory.^13^ The number of secondary infections from a primary infection were represented by a negative binomial distribution with two parameters: the mean number of secondary infections, R, and a dispersion parameter, k, which varies inversely with the heterogeneity in infectiousness among individuals. The negative binomial probability distribution for the number of cases generated in an infectious disease outbreak with either R < 1 or R > 1 has been previously derived.^14^^,^ 15 In the case of R < 1, stuttering chains of transmission may occur but there should be no major outbreaks. When R > 1, a proportion of primary cases should give rise to a sustained transmission chain, although some primary cases will fail to trigger a major outbreak due to stochastic extinction.

With a negative binomial offspring distribution the probability (\begin{equation*}h_i\end{equation*}) of a transmission chain having an overall size of \begin{equation*}i\end{equation*} is:

\begin{equation*}h_{i} = \frac{\Gamma (ki+i-1)}{\Gamma(ki) \Gamma(i+1)} \frac{(\frac{R}{k})^{i-1}}{(1+\frac{R}{k})^{ki+i-1}},\%0A\end{equation*}

where Γ represents the gamma function. Equation (1) can then be used to generate a probability density function of final outbreak sizes for particular values of R and k. Maximum likelihood estimates for *R* and *k* were found using the probability generating function and the likelihood of the observed final size distribution

\begin{equation*}L = \prod_{i=1}^{\infty} h_{i}^{n_{i}},\end{equation*}

where \begin{equation*}n_i\end{equation*} is the number of chains of size \begin{equation*}i\end{equation*} that are observed. The largest outbreak size observed varied from 149 to 160 cases in the different introductions scenarios, therefore we truncated the probability density function at *i*=149 and calculated the probability of observing an outbreak of ≥149 cases. The ML estimates of *R* and *k* were obtained by minimizing the negative log likelihood of both parameters simultaneously and confidence intervals for these parameter estimates were then calculated by creating likelihood profiles.^16^ For several of our transmission chain scenarios we could not obtain a confidence interval for *k* as the estimate of \begin{equation*}k \rightarrow \infty\end{equation*}. We estimated *R* and *k* assuming either perfect surveillance or a 25--50% probability of case detection where the probability of a case being detected was either unbiased or depended on the outbreak size. In the first imperfect surveillance scenario all cases (primary or secondary) have an equal 25 or 50% probability of being detected. In the second surveillance scenario primary cases have only a 25 or 50% probability of being detected, but all secondary cases in an already identified outbreak are detected.

Because the majority of the confirmed cases in Mexico in 2013 have been from Huejutla, we also calculated the initial epidemic growth rate (*r*) for Huejutla based on the weekly progression of cases up to the peak of the outbreak in that city. Assuming that the growth rate was exponential during the first three weeks of the outbreak, *r(t) = log (N) / t *for *N* cumulative cases at time t. The decline in cases from Huejutla after the peak during the week of 9/29 may have been due to control efforts that reduced the final outbreak size, leading to an underestimate of *R* in the absence of control. To estimate *R_0_* in the absence of control we calculated *R* based on the value of *r* during the first three weeks of the outbreak in Huejutla. When *r*>0 as it was in Huejutla, the basic reproductive number is bounded by 1 < *R* < *e^rT_c_^* where T_c _is the generation interval.^28^ If we model cholera transmission dynamics using an SIR model with individuals leaving the infectious class at rate *a*, then the generation interval is specified by an exponential distribution with a mean *T_c_=1/a *and *R = 1 + rT_c_*.^28^ We calculated *R = 1 + rT_c_* and *R < e^rT_c_^* assuming that *T_c_*=3, 5, or 10 days to set upper and lower bounds on* R* during the initial phase of the Huejutla outbreak. We also calculated the probability of observing outbreaks of the size seen in Huejutla given our estimated values of R and k by simulating 100,000 transmission chains using the negative binomial model.

## Results

The estimate of *R* varies slightly based on the assumed number of individual transmission chains in the recent Mexico cholera outbreak. Assuming perfect surveillance under our two most likely scenarios, the maximum likelihood (ML) estimate for *R*=1.04 (95% CI: 0.85—1.55; Figure 3a) with an ML estimate for *k*=4.5 (95% CI: 0.3—∞) if assume a maximum generation interval of one week (13 transmission chains). For a maximum generation interval of two weeks (8 transmission chains) the estimate increases to *R*=1.07 (95% CI: 0.89—1.34; Figure 3c) and *k*=2984 (95% CI: 0.8—∞). In both cases the 95% confidence interval for R crosses 1, indicating that conditions in Mexico may be close to those needed to sustain larger epidemics. Under the scenario with the highest number of individual transmission chains the ML estimate for *R*=0.80 (95% CI: 0.05—∞) and *k*=0.0039 (95% CI: 0.00014—0.0315). Under this scenario* R* is lower because several larger transmission chains are split into separate transmission chains without a secondary case. Under the scenario with the fewest transmission chains the ML estimates are *R*=1.12 (95% CI: 0.91—1.46) and *k*=22028 (95% CI: 6128—∞).

Relaxing the assumption of perfect surveillance and assuming that 50% of cases go undetected independent of outbreak size lowers most *R* estimates slightly. Under the scenario with the highest number of individual transmission chains the ML estimate for* R*=0.73 (95% CI: 0.04—∞) and *k*=0.002 (95% CI: 0.00009—0.020), while for the scenario with the fewest transmission chains the ML estimates are *R*=1.08 (95% CI: 0.92—1.68) and *k*=94000 (95% CI: 0.15—∞). For the likely scenario with a maximum generation interval of one week, *R*=1.01 (95% CI: 0.86—1.34) and *k*=107 (95% CI: 0.27—∞), and for the likely scenario with a maximum generation interval of two weeks, *R*=1.05 (95% CI: 0.90—1.29) and *k*=2437 (95% CI: 0.42—∞). If only 25% of cases are detected then *R* estimates decrease further. Under the scenario with the highest number of individual transmission chains the ML estimate for R= 0.58 (95% CI: 0.03—∞) and k=0.0015 (95% CI: 0.00006—0.014), while for the scenario with the fewest transmission chains the ML estimates are* R*=1.05 (95% CI: 0.91—1.51) and* k*=4.2e+6 (95% CI: 0.10—∞). For the likely scenario with a maximum generation interval of one week, *R*=0.98 (95% CI: 0.87—1.12) and *k*=1723 (95% CI: 0.24—∞), and for the likely scenario with a maximum generation interval of two weeks,* R*=1.02 (95% CI: 0.90—1.16) and *k*=2363 (95% CI: 0.32—∞). If we assume detection is not independent but depends on outbreak size, such that 50 or 75% of primary cases go undetected while secondary cases in an identified outbreak are detected, detection of only 25% or 50% of primary cases lowers most of our *R* estimates while also increasing the uncertainty in the estimates. With 50% detection of primary cases in the scenario with the highest number of individual transmission chains the ML estimate for *R*=0.67 (95% CI: 0.03—∞) and* k*=0.0020 (95% CI: 0.00008—0.015), while for the scenario with the fewest transmission chains the ML estimates are *R*=1.10 (95% CI: 0.89—2.61) and *k*=28.6 (95% CI: 0.09—∞). For the likely scenario with a maximum generation interval of one week, *R*=1.04 (95% CI: 0.79—1.74) and *k*=0.85 (95% CI: 0.145—∞), and for the likely scenario with a maximum generation interval of two weeks, *R*=1.05 (95% CI: 0.87—1.56) and *k*=421 (95% CI: 0.255—∞). With only 25% detection of primary cases in the scenario with the highest number of individual transmission chains the ML estimate for *R*=0.50 (95% CI: 0.02—∞) and *k*=0.0012 (95% CI: 0.00005—0.008), while for the scenario with the fewest transmission chains the ML estimates are* R*=1.13 (95% CI: 0.84—3.70) and *k*=1.03 (95% CI: 0.047—∞). For the likely scenario with a maximum generation interval of one week, *R*=0.99 (95% CI: 0.72—1.74) and *k*=0.42 (95% CI: 0.088—∞), and for the likely scenario with a maximum generation interval of two weeks, *R*=1.03 (95% CI: 0.82—1.68) and *k*=2.31 (95% CI: 0.136—∞).

**Maximum likelihood profiles for R and k.**
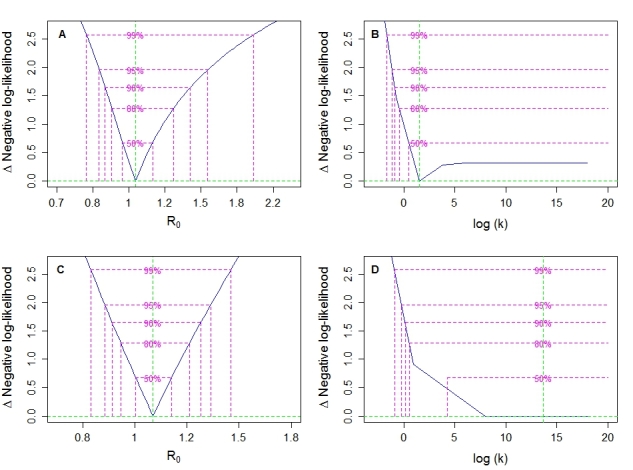
Maximum likelihood profiles for (a) *R* and (b) *k* for the likely introduction scenario with a maximum generation interval of one week and 13 transmission chains. (c-d) *R* and *k* estimates for scenario with a maximum generation interval of two weeks and 8 transmission chains.

The probability of observing an outbreak as large as the 150+ case outbreak observed in Huejutla is only 0.32% when *R*=0.80 and *k*=0.0039 (ML estimates from maximum introductions scenario with perfect surveillance), but increases to 21.3% when *R*=1.12 and *k*=3.62 (ML estimates from minimum introductions scenario). For values of* R*<1, lower *k* values increase the probability of observing larger outbreaks, but when *R*>1, lower *k* values decrease the probability of the largest outbreaks. The initial growth rate (*r*) through the first three weeks of the outbreak in Huejutla was 0.235 day^-1^. Assuming a mean generation interval (*T_C_*) of 5 days the estimate of *R* in Huejutla varies from *R = 1 + rT_C_* = 2.18 to *R = e^rT_C_^*= 3.24. If *T_C_*=3 days then the range for *R *= 1.71—2.02, while if *T*_C_=10 days the range for *R*=3.35—10.49.

**Simulated frequency distributions of outbreak sizes.**
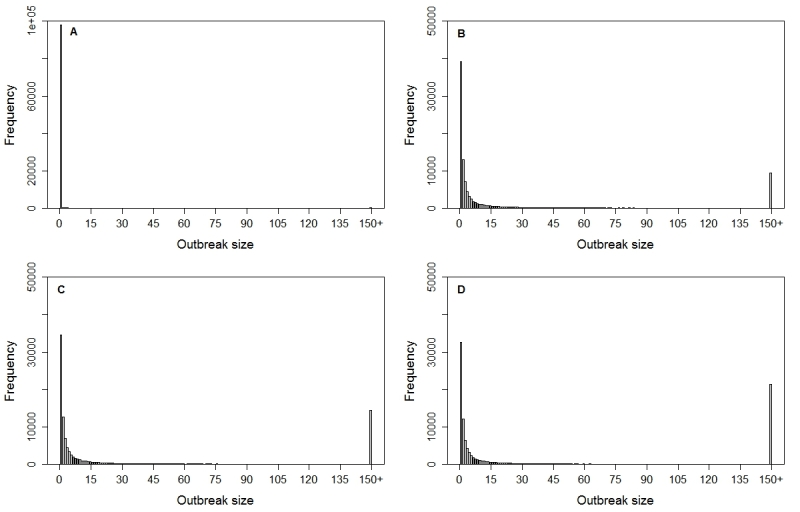
Simulated frequency distributions of final outbreak sizes for the ML estimates of R and k obtained under four scenarios (a) a maximum of 37 introductions (*R*=0.80, *k*=0.004), (b) a likely scenario of 13 introductions with a maximum generation interval of one week (*R*=1.04,* k*=4.5), (c) a likely scenario of 8 introductions with a maximum generation interval of two weeks (*R*=1.07, *k*=2984), or (d) a minimum of 5 introductions (*R*=1.12, *k*=22028).

## Discussion

We estimate *R* for the recent cholera outbreak in Mexico to be very close to one, suggesting that the efficiency of cholera transmission in Mexico is close to that necessary for a large epidemic to occur. The estimate of *R* assuming perfect surveillance was greater than one except under the scenario where number of potential primary cases was maximized. The estimate of *R* was not particularly sensitive to the under detection of up to 75% of cases. However, if only 25% of cases were reported then the best estimate of *R* decreases to just below one (0.98 or 0.99) for the likely scenario with a maximum generation interval of one week. The initial epidemic growth rate of 0.23 day^-1^ observed in Huejutla, Hidalgo also indicates that under the right conditions cholera could spread rapidly in Mexico, even if sustained transmission is unlikely due to the implementation of control measures or the diminishing of temporarily favorable environmental conditions.

The majority of cases occurred in the municipality of Huejutla, suggesting that there is significant spatial heterogeneity in *R* within Mexico. Estimation of *R* based on the initial growth rate in Huejutla also indicates that it is likely that *R* was significantly higher than one in this city before control measures were implemented. The failure of introductions in the states of Veracruz, San Luis Potosi, and Mexico to spread suggests that *R*<1 in most regions of Mexico. One might assume that such heterogeneity could also result from super spreading events in a population with a generally low *R*, but our low dispersion estimates (i.e., high estimates of *k*) indicate this may not be the case; however, there are wide confidence intervals on these estimates and in most cases the data was insufficient to properly estimate *k*. In addition, when the assumption of perfect surveillance was relaxed, reducing the probability of case detection led to smaller estimates of *k* under certain scenarios.

According to UNICEF, 94% of Mexico’s population has some access to improved drinking water and 85% have access to improved sanitation, compared to regional averages of 94% and 82% respectively.^17^^,^ 18 However, country averages do not paint the full picture, and some areas may have poor overall water and sanitation. Hidalgo, the Mexican state where most cases have occurred, has average access to potable water and below average sanitation, although a number of states have worse records.^19^ Huasteca, the region of Hidalgo where the majority of cases have occurred, lags behind the rest of the state in health services, water services and electricity.^20^ Due to the geographic heterogeneity in vulnerability observed during this cholera outbreak, it is important to identify the most vulnerable areas in Mexico and neighboring countries. It is likely that other communities are at risk of experiencing an outbreak as large or larger than the one experienced in Huejutla following an introduction.

The beginning of the epidemic in September 2013 aligned with both a tropical storm and hurricane that caused heavy rains, floods, landsides and population displacement in the region.^21^^,^ 22 ^,^ 23 However, it is not known how these storms affected the specific communities that were most affected by cholera. Flooding and heavy rains may have increased the *R* of cholera in these communities during the rain and flooding or immediately thereafter. If conditions for cholera were temporarily elevated by environmental conditions then the quick decline in cases following the peak of the outbreak may have resulted from a natural improvement in sanitation and access to clean drinking water following the cessation of flooding rather than control measures.

This analysis was limited by a lack of detailed knowledge of the number and specific locations of both for cases introduced into Mexico and the secondary cases they cause. Some of the information regarding the identity of municipalities with confirmed cases on informal sources such as newspaper reports. Better information on introductions and the resulting specific transmission chains would help to clarify the analysis, particularly because the estimate of *R* was above or below one depending on how many separate transmission chains we assumed occurred during the outbreak. Furthermore, the approaches used are based on a model of person-to-person transmission; but cholera can also spread through contaminated environmental sources.^24^ Hence, these preliminary results must be interpreted with caution.

There are many regions in the Americas with worse infrastructure than Hidalgo State in Mexico. During the previous cholera epidemic in Mexico, the state of Hidalgo had a cumulative incidence rate from 1991-1996 (70.5 cases per 100,000) that was higher than the national average. However, several states had incidence rates that were much higher, including Campeche (614 per 100,000), Yucatan (290 per 100,000), Tabasco (217 per 100,000) and Chiapas (146 per 100,000).^25^ Although access to improved water and sanitation has increased in all of these states since 1991, their high incidence rates relative to Hidalgo in the 1990s suggest that they could be at a greater risk of a major cholera outbreak if it is introduced into one of these states. A recent review of the potential for the re-emergence of cholera throughout Latin America and the Caribbean also shows that many countries in the region have a lower access to basic services and infrastructure than Mexico and are therefore likely more susceptible to a major epidemic.^26^ If there is evidence of sustained transmission, countries may consider applying to the newly established oral cholera vaccine stockpile to control or prevent a major epidemic.^27^

Frequent travel makes additional introductions likely, and our results suggest many areas are likely on the cusp of being able to support sustained cholera transmission, if they cannot already. However, it is important to remember that *R* for cholera is a modifiable factor. Heavy rain or other climatic factors could temporarily improve conditions for cholera transmission in the affected regions. Likewise, careful monitoring and public health interventions can help to keep *R* below one and head off any nascent epidemics. So far, Mexico appears to have done well in this regard. To avoid further expansion of cholera in the Americas, other countries must follow their example and remain vigilant.

## Authorship

Corresponding author: Justin Lessler, jlessler@jhsph.edu

*Sean M. Moore and Kerry L. Shannon contributed equally to this manuscript
